# Novel Therapies for Prosthetic Joint Infections Caused by Methicillin-Resistant *Staphylococcus aureus*

**DOI:** 10.3390/pathogens14111102

**Published:** 2025-10-29

**Authors:** Xi Xiang, Xin Jin, Qi Yang, Lili Zou, Yueqing Wang, Tianxu Wang, Xun Sun

**Affiliations:** 1 The Third Clinical Medical College of the China Three Gorges University, Gezhouba Central Hospital of Sinopharm, Yichang 443000, China; xiangxi28@163.com (X.X.);; 2 Yichang Key Laboratory of Infection and Inflammation, College of Basic Medical Science, China Three Gorges University, Yichang 443000, China; 3 Hubei Key Laboratory of Tumor Microenvironment and Immunotherapy, College of Basic Medical Science, China Three Gorges University, Yichang 443000, China

**Keywords:** prosthetic joint infection, methicillin-resistant *Staphylococcus aureus*, enhanced therapy, new therapy

## Abstract

Periprosthetic joint infection (PJI) is a serious complication following total joint replacement, with methicillin-resistant *Staphylococcus aureus* (MRSA) being the primary pathogen. The treatment challenges posed by MRSA’s antibiotic resistance further highlight the critical importance of research in this field. Current antibiotic therapies for periprosthetic joint infection caused by methicillin-resistant *Staphylococcus aureus* (MRSA-PJI) are limited by considerable side effects, such as high costs and the development of resistance. Therefore, there is an urgent need to explore novel alternative or adjunctive therapies. This review provides a comprehensive overview of several innovative therapeutic strategies. These include monoclonal antibody therapies that target specific bacterial components; phage therapy, which can either independently or synergistically degrade biofilms and enhance antimicrobial efficacy, characterized by its high specificity; antimicrobial peptides, capable of disrupting bacterial membrane integrity and exhibiting dual antibiofilm activity, with a reduced tendency to induce resistance; and nanoparticles and hydrogels, which function as drug delivery systems for sustained release, thereby improving both preventive and therapeutic outcomes. However, these novel therapies also face challenges such as high production costs and limited stability, underscoring the need for further research and optimization. Future efforts should focus on additional studies, clinical trials, and the development of robust regulatory frameworks to fully realize the potential of these treatments for MRSA-PJI.

## 1. Introduction

Total joint replacement (TJR) is considered one of the most successful surgical interventions in medical history and remains the optimal treatment for end-stage large joint diseases. The number of such surgeries is expected to double in the next decade [1]. However, the rising volume of total joint arthroplasty is associated with a corresponding increase in the incidence of postoperative complications [2].

Periprosthetic joint infection (PJI) is one of the most severe complications following TJR and represents a major challenge in contemporary orthopedics [3]. According to a recent retrospective study performed in the United States from 2001 to 2009, the relative incidence of PJI ranged between 2.0% and 2.4% of total hip arthroplasties and total knee arthroplasties and increased over time [4]. A key feature of PJI is the presence of bacterial biofilms on the joint prosthesis, and the core of clinical treatment focuses on eradicating the biofilms formed by bacterial infections [5,6]. Research has shown that methicillin-resistant *Staphylococcus aureus* (MRSA) is one of the leading pathogens responsible for PJI, accounting for 25% of all pathogenic microorganisms involved [7]. Its multidrug-resistant characteristics significantly complicate treatment, leading to high recurrence rates and prosthesis removal rates. Furthermore, MRSA is also a significant risk factor for multiple artificial joint heterotopic PJIs [8].

**Figure 1:**
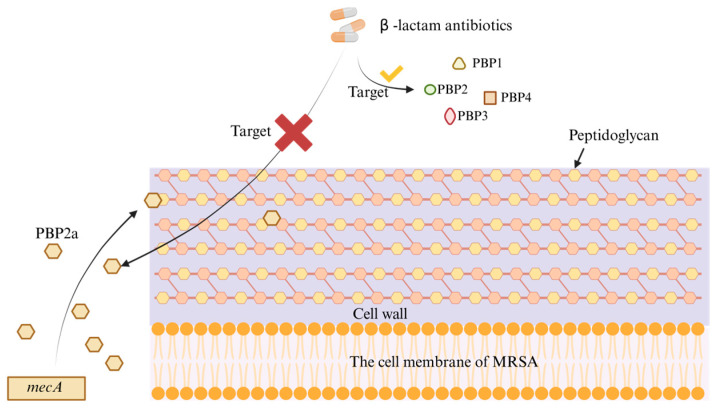
The mechanism of MRSA resistance. PBP2a, encoded by the *mecA* gene, has low affinity for β-lactam antibiotics, making it ineffective as a target for these drugs.

*Staphylococcus aureus* is a predominant opportunistic pathogen in both healthcare and community environments and is implicated in a broad spectrum of clinically relevant infections, ranging from superficial skin conditions to severe invasive diseases [9]. Since the 1960s, MRSA has emerged and spread globally, becoming a major cause of bacterial infections [10]. The emergence of MRSA is due to the acquisition of the *mec* gene on the *Staphylococcus* chromosome (SCC *mec*), a mobile genetic element capable of encoding either the *mecA* or *mecC* genes [11] (Figure 1). The penicillin-binding protein 2a (PBP2a) encoded by the *mecA* gene is an enzyme responsible for cross-linking the peptidoglycan in bacterial cell walls. Unlike the PBPs produced by methicillin-sensitive *Staphylococcus aureus* (PBP1, PBP2, PBP3, and PBP4), PBP2a produced by MRSA has a lower affinity for β-lactam antibiotics, making it ineffective as a target for these drugs (penicillin, cephalosporin and carbapenems) [12]. PBP2a replaces the biosynthetic functions of other PBPs, allowing cell wall synthesis to continue and enabling the bacteria to evade the bactericidal and lytic effects of antibiotics [13]. MRSA causes severe infections, and due to the high incidence and mortality and its antibiotic resistance, the treatment of MRSA infections has become a pressing global public health issue [14].

The treatment of MRSA-induced PJI (MRSA-PJI) primarily involves two approaches: antibiotic therapy and surgical intervention. Surgical procedures, while effective, cause significant physical trauma to patients and increase their financial burden to a certain extent. Additionally, the failure rate of PJI treatment remains high even with the use of one- or two-stage revision surgery following prosthetic joint replacement [15]. These challenges underscore the urgent need for non-surgical therapeutic alternatives. Currently, the most commonly used antibiotics for MRSA-PJI are vancomycin, linezolid, and daptomycin [16,17,18,19,20]. However, these traditional targeted antibiotics have significant drawbacks, including high costs, toxic side effects, complex treatment regimens, and the exacerbation of resistance development, all of which negatively impact patient prognosis. The development and approval for marketing of new antibiotics is costly and eventually subject to the onset of resistance. Therefore, the exploration of novel alternative or adjunct therapies is necessary.

**Figure 2:**
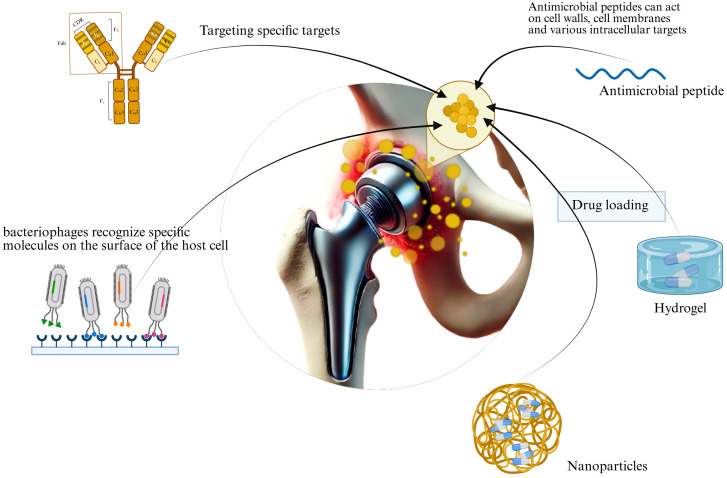
Schemes of new therapies for MRSA-PJI. The new therapy consists of two parts: new drugs and new enhanced therapies. New drugs include: monoclonal antibodies, bacteriophages, and antimicrobial peptides. New enhanced therapies include nanoparticle delivery and hydrogel delivery.

This review provides a thorough and critical analysis of current literature on emerging therapies for MRSA-PJI (Figure 2), with the aim of fostering innovation in their clinical application and further development. The significance of these novel therapeutic strategies extends well beyond clinical implementation. Not only can these novel strategies offer opportunities for healthcare professionals to refine existing treatment protocols or introduce groundbreaking approaches for eradication of MRSA-PJI, but they also hold the promise of improved cure rates, better long-term outcomes, enhanced quality of life, and reduced socioeconomic burdens for patients. Moreover, the successful translation of these therapies may effectively integrate foundational research with clinical applications, thereby stimulating interdisciplinary innovation across areas including molecular medicine, clinical infectiology, pharmaceutical sciences, and biomaterials engineering.

## 2. Novel Therapeutics

### 2.1. Monoclonal Antibody Therapy

Therapeutic monoclonal antibodies (mAbs) typically belong to the class of γ-immunoglobulins (IgG). IgG is a Y-shaped 150 kDa immunoglobulin composed of two identical heavy and light chains linked by disulfide bonds [21]. The variable regions of each heavy and light chain form antigen-binding sites, known as the antigen-binding fragments (Fab), while the crystallizable fragment (Fc), responsible for the effector function, is composed of two constant structural domains. The antibody selectively binds to the antigen via its variable region, playing a pivotal role in its pharmacological function. Due to its pH-dependent recycling via the neonatal Fc receptor, this bivalent IgG molecule exhibits a long serum half-life.

The mechanism of monoclonal antibodies against MRSA primarily involves specific targeting, localization, bacterial killing, and toxin neutralization. For example, the monoclonal antibody MEDI4893* targeting α-toxin binds with high affinity to a critical overlapping epitope on the α-toxin, specifically blocking the assembly of the monomer into the heptameric pore structure. This disrupts the critical steps in pore formation, reducing the damage caused by the toxin to lung and immune cells and thereby mitigating the severity of the infection [22]. In a model of influenza-associated MRSA pneumonia, MEDI4893* administered five days after influenza infection (24 h prior to MRSA infection) significantly improved survival rates when used alone, with even greater efficacy when combined with the anti-influenza hemagglutinin mAb FY1 [23].

**Figure 3:**
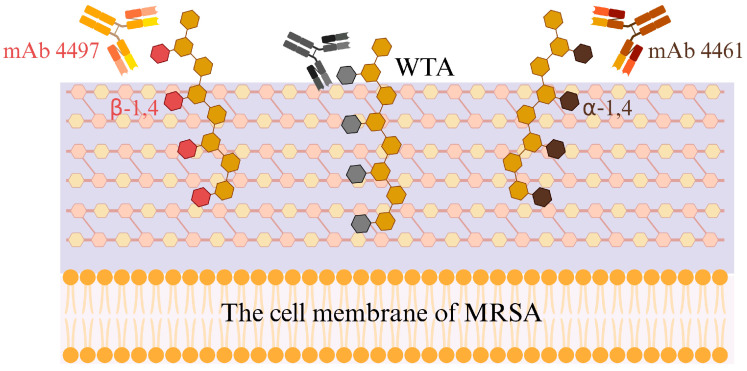
Schematic diagram of the mechanism of AMPs against WTA.

Monoclonal antibodies, such as mAb4461 and mAb4497, directed against wall teichoic acid (WTA) in *Staphylococcus aureus*, specifically recognize and bind to glycosylated GlcNAc residues on the WTA structure. These antibodies recognize the sugar moieties, phosphate groups, and ribitol phosphate backbone units of WTA, thereby binding to the bacterial surface (Figure 3). mAb4461 preferentially binds to the internal sugar moiety of α-1,4-GlcNAc-modified WTA, while mAb4497, due to the flexibility of the ribitol phosphate backbone, cross-recognizes β-1,3/β-1,4-GlcNAc-modified WTA [24]. Wang developed a conjugate by cross-linking the antigen-binding fragments of monoclonal antibodies against WTA with poly-sialic acid (PSA), forming mAbs-PSA conjugates. This conjugate effectively targets and induces calcification on the surface of MRSA, eliminating the bacteria by disrupting MRSA’s energy metabolism and several essential metabolic pathways. Furthermore, the bacterial calcification leads to increased expression of the calcium-binding proteins S100A8/S100A9 in macrophages and monocytes, triggering the activation of these cells to an inflammatory state, thereby promoting bacterial eradication [25].

Monoclonal antibody therapy has demonstrated significant progress in combating MRSA-PJI. Bruce developed a diagnostic-therapeutic monoclonal antibody by conjugating 4497-IgG1 with a radioactive isotope [26]. This mAb specifically targets the site of infection, assisting in imaging while simultaneously binding to the MRSA biofilm and eliminating bacteria via alpha radiation (e.g., bismuth-213). In addition to the aforementioned monoclonal antibody, several others targeting MRSA are currently under investigation.

Based on these studies, monoclonal antibodies exhibit high target specificity, a favorable safety profile, and broad therapeutic windows, underscoring their potential for clinical use in MRSA treatment. Further evaluation in in vivo models of MRSA-PJI is warranted to accelerate their translation from basic research to clinical practice, thereby expanding the range of effective therapeutic strategies for MRSA-PJI. Monoclonal antibodies are commonly delivered to the periprosthetic tissue and mature biofilms of MRSA via intravenous injection [26,27,28], where they specifically accumulate on infected joint prostheses over time [26]. Although antibodies may exhibit high specificity, the required dosage for effective treatment is typically large [29]. Subcutaneous administration of monoclonal antibodies, however, is hindered by low bioavailability and potential immunogenicity. Furthermore, Bruce van Dijk et al. reported that the biological half-life of free monoclonal antibodies reaches a plateau between 24 and 72 h [26]. In contrast, liposome-encapsulated monoclonal antibodies exhibit sustained release properties, with continuous release observed even at 42 days [30]. Moreover, liposome-encapsulated monoclonal antibodies effectively protect the subcutaneously administered antibodies from degradation. Future work should focus on further optimizing liposomal formulations to enhance their loading capacity, stability, and release kinetics.

### 2.2. Phage Therapy

**Figure 4:**
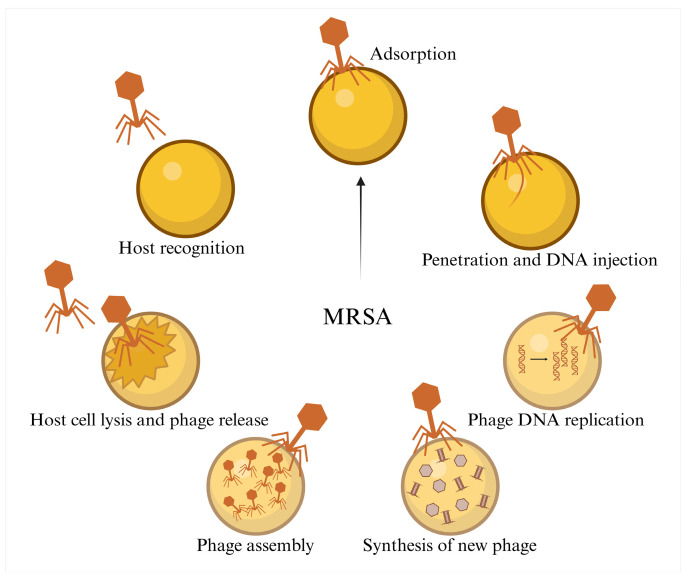
The mechanism of phages in combating MRSA.

Bacteriophages are viruses with all the common characteristics of viruses and are widely found in various environments [31,32]. The core mechanism of phage therapy against MRSA in the treatment of PJI involves the specific binding of phage tail proteins to bacterial surface receptors, such as the cell wall proteins and teichoic acids of MRSA, or the capsular polysaccharides of PJI-associated bacteria. This binding facilitates the injection of genetic material into the bacterial cell, hijacking the bacterial metabolic system to replicate the phage. Ultimately, the phage mediates bacterial lysis via endolysins (peptidoglycan-hydrolyzing enzymes) and holins (disrupting the cell membrane), releasing progeny phages that continue to infect the surrounding bacteria [33] (Figure 4). Unlike antibiotics, bacteriophages can self-amplify at the site of infection, providing sustained bacterial clearance [34]. One of the therapeutic challenges in treating MRSA-PJI is the formation of biofilms on joint prosthetics, which increases antibiotic resistance. Some phages contain polysaccharide depolymerases in their tail structures, which can degrade the extracellular matrix of biofilm-associated bacteria, effectively disrupting the biofilm and aiding in the colonization of phages around the MRSA biofilm on the prosthetic [35,36].

Phage therapy shows particular promise in treating MRSA-PJI. Zhong isolated a bacteriophage targeting MRSA, called StAP1 [37], which can effectively infect all SCC *mec* types present in MRSA strains and has shown significant therapeutic efficacy in a murine model of MRSA infection. In a separate study, researchers showed that bacteriophage ØK, which possesses anti-MRSA activity, could be successfully loaded onto orthopedic carbon scaffolds and continuously released over a period of seven days [38]. Additionally, a novel inhalable dry powder formulation has been developed, consisting of porous PLGA microspheres encapsulating heat-resistant phages conjugated with indocyanine green [39]. This formulation has demonstrated potent bactericidal effects against MRSA. Phage therapy is generally well-tolerated. In a treatment study involving 20 patients with drug-resistant mycobacterial infections, no adverse reactions were observed as a result of phage therapy, regardless of the pathogen, the administered phage, or the delivery route [40]. In a Phase 1 clinical trial, researchers administered a phage mixture (AB-SA01) intranasally to nine patients with persistent chronic sinusitis who tested positive for *Staphylococcus aureus*. The phage therapy was well tolerated, and no deaths were recorded in any group [41]. Furthermore, studies have shown that infection eradication was observed in nine patients, indicating that the treatment is effective.

Moreover, many studies have shown that combining phages with antibiotics results in synergistic effects, reducing the minimum inhibitory concentration (MIC) of resistant strains and improving bacterial clearance efficiency [42,43,44]. For instance, Coyne investigated the synergy between the Romulus bacteriophage and humanized doses of daptomycin and cefazolin. The results demonstrated that the combination of phage and antibiotics exhibited bactericidal activity against MRSA strains without increasing antibiotic resistance post-treatment [45].

Phages offer a promising therapeutic strategy against MRSA-PJI. Currently, direct phage delivery (including intravenous administration and local application) remains the most extensively studied form of phage therapy, particularly in the context of prosthetic joint infections. Compared to antibiotics, phages, when used in combination with antibiotics, not only help eradicate the infection but also prevent its persistence and re-infection [34]. In a murine model of MRSA-induced implant-associated infections, local administration of phages in combination with antibiotics provided sustained effects and eradicated MRSA by day 10 [46]. Additionally, certain phages are capable of degrading biofilms, addressing a core challenge in the treatment of MRSA-PJI. Phage therapy not only has fewer adverse reactions but also exerts less negative impact on the human microbiota compared to antibiotics, which often disrupt the microbial community extensively. Current research has sufficiently demonstrated the potential of phage therapy in treating MRSA-PJI.

### 2.3. Antimicrobial Peptide Therapy

Antimicrobial peptides (AMPs) are a class of small peptides typically composed of 12 to 50 amino acid residues [47,48]. As biologics, AMPs, identified in a wide range of animal, insect, and plant species, function as host defense peptides and are essential components of innate immunity [49]. AMPs, which have antibiofilm and immunomodulatory properties, are less prone to induce resistance, positioning them as promising candidates for the next generation of antimicrobial agents [48,50]. AMPs possess a complex mechanism of action, targeting the cell wall, cell membrane, and various intracellular components, as well as inhibiting biofilm formation and modulating host immune system activity [51].

AMPs have shown promising efficacy in combating MRSA and have been applied in animal models of PJI. GN1 is a potent and non-toxic antimicrobial peptide that counteracts resistance mechanisms in MRSA by inhibiting both the production and enzymatic activity of key resistance determinants. By reversing these resistance traits, GN1 restores the susceptibility of MRSA to low concentrations of multiple β-lactam antibiotics [52]. Additionally, Songnaka isolated a novel antimicrobial peptide from the soil bacterium *Brevibacillus sp. SPR-20*, which inhibits MRSA at concentrations ranging from 2–32 μg/mL by inducing bacterial membrane damage [53]. Pavel demonstrated, in animal models, that embedding antimicrobial peptides into bone cement effectively prevents MRSA attachment and inhibits over 80% of biofilm formation on the implant surface [54]. In a rabbit PJI model infected with *Staphylococcus aureus*, the antimicrobial peptide PR-39 exhibited strong antibacterial effects [55]. On days 7 and 14 post-PJI, the treated group showed significantly reduced erythrocyte sedimentation rates and C-reactive protein levels compared to the control group, which did not receive antimicrobial peptide treatment.

These examples highlight that AMPs possess broad-spectrum antibiofilm activity, making them highly suitable for the treatment of MRSA-PJI. Moreover, AMPs can synergize with antibiotics, reducing dosage and toxicity. Given their potential for structural modification, AMPs offer great promise for optimizing their activity and stability. Antimicrobial peptide therapy shows broad potential for the treatment of MRSA-PJI, and future research should focus on in vivo testing of AMPs in clinically representative MRSA-PJI models.

## 3. Emerging Therapeutic Approaches

### 3.1. Nanoparticles

Nanoparticles are materials with particle sizes ranging from 1 to 100 nanometers in diameter [56]. Due to their high surface-to-volume ratio, nanoparticles exhibit a variety of novel properties, including mechanical, chemical, electrical, optical, and magnetic characteristics. These properties enable nanoparticles to target multiple bacterial sites, thereby enhancing their antimicrobial efficacy against both resistant and susceptible bacteria [57]. Furthermore, nanoparticle-based materials can serve as carriers to improve the bioavailability and effectiveness of antibiotics [58].

**Figure 5:**
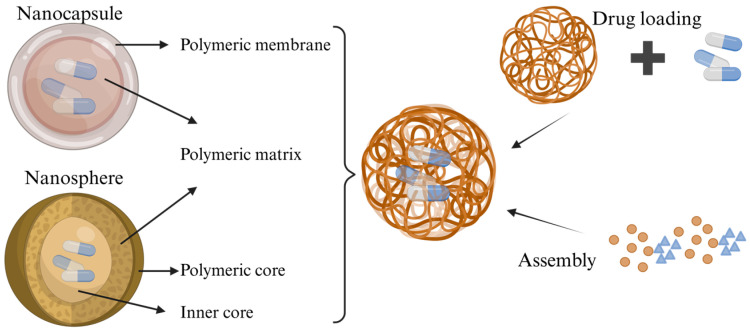
Synthesis strategies of nanoparticle polymers.

Nanoparticles encompass both nanocapsules and nanospheres, which differ in their morphology. Nanocapsules consist of an oily core in which the drug is typically dissolved, surrounded by a polymer shell that regulates the release profile of the drug from the core [59]. Nanospheres, on the other hand, are based on a continuous polymer network, within which the drug may be retained internally or adsorbed onto the surface. When used as carriers, strategies for synthesizing nanoparticle polymers include post-loading and co-loading approaches [60] (Figure 5).

Nanoparticles have demonstrated exceptional performance as delivery systems in combating MRSA infections. Guevara reported a formulation of PLGA nanoparticles loaded with antibiotics, which can induce sustained and controlled antibiotic release, resulting in prolonged antimicrobial activity against MRSA and *Pseudomonas aeruginosa*. This controlled and sustained antimicrobial effect is precisely what is urgently needed for the prevention and treatment of MRSA-PJI. Beyond its use in post-infection treatment, local delivery following joint replacement surgery can enable the sustained release of antibiotics around the prosthetic joint, offering a preventative strategy against the development of MRSA-PJI. In animal models, nanoparticles triggered by infection microenvironments, along with thermal-enhanced chemokinetic therapy and M1 macrophages, were effective in controlling MRSA-induced infections [61]. These findings highlight the promising potential of nanoparticles as a new enhancement therapy, demonstrating remarkable prospects in MRSA treatment. However, nanoparticle therapy carries the potential for adverse local tissue reactions. For instance, nanoparticles are prone to recognition and uptake by the mononuclear phagocyte system (MPS), leading to localized toxicity in organs such as the liver and spleen [62]. Macrophages in the liver, spleen, and bone marrow, which constitute the core of the MPS, are the primary sites for nanoparticle clearance. Upon intravenous injection, serum proteins (e.g., opsonins) adsorb onto the surface of nanoparticles, which are then recognized and internalized by scavenger receptors on macrophages, causing the particles to be cleared from circulation. Nanoparticles that are unPEGylated, charged (especially negatively charged), or possess abnormal sizes (either 250 nm) are more readily cleared by the MPS [63]. The half-life of nanoparticles is primarily determined by their physicochemical properties (e.g., particle size, surface modifications, composition) and the route of administration. The core function is to enhance accumulation in target tissues (such as tumors) by prolonging circulation time. For example, nanoparticles within the 10–200 nm size range can avoid renal filtration while reducing recognition and clearance by the MPS (macrophages in the liver and spleen). Particles larger than 200 nm may activate the complement system, triggering rapid clearance. Thus, the 10–200 nm range is optimal for achieving extended half-lives [64]. As research progresses in understanding the impact of nanoparticle size on interactions with the human body, the occurrence of adverse local tissue reactions can be minimized. Researchers are advancing towards establishing nanoparticles as a standard tool for clinical diagnostic imaging and drug delivery.

### 3.2. Hydrogels

Hydrogels have emerged as pivotal drug delivery vehicles owing to their high water content, excellent biocompatibility, and three-dimensional network structure. These attributes not only improve drug stability but also enable sustained release profiles through strong interfacial adhesion. Stimuli-responsive hydrogels facilitate precise and targeted drug release, allowing localized delivery to specific sites such as areas of inflammation or the gastrointestinal tract. This site-specific approach markedly reduces systemic toxicity [65].

The use of hydrogels as drug delivery systems has shown promise in addressing MRSA infections. Researchers reported a copper-containing chitosan hydrogel that, when applied in 3D-printed scaffolds, significantly accelerated bone repair and eradicated MRSA-associated infections in both the cranial bone defect repair model and the ectopic MRSA infection model in Sprague-Dawley rats [66]. Another hybrid antimicrobial gel, designed for chronic wound healing, not only promoted cell proliferation and macrophage polarization to the M2 phenotype but also exhibited strong antibacterial activity against Gram-positive bacteria. This hydrogel accelerated skin regeneration in diabetic and burn patients with MRSA infections by enhancing the regenerative responses of M2 macrophages, reducing inflammation, and promoting angiogenesis [67]. Tu developed a multifunctional hydrogel by crosslinking hyperbranched poly(L-lysine)-modified manganese dioxide nanocatalysts with hydrophilic polymer (PEGMA-co-GMA-co-AAm), which effectively inhibited bacterial quorum sensing systems, downregulated virulence genes, and disrupted bacterial metabolism. This hydrogel killed 94.1–99.5% of MRSA even at 10^9^ CFU/mL [68]. Additionally, a FABA hydrogel, made by self-crosslinking Si-Ca-Cu nanoglass modified with F127-CHO (FA) and sodium alendronate, significantly inhibited MRSA growth in vitro, while demonstrating excellent cell and blood compatibility [69]. Furthermore, a multifunctional hydrogel wound dressing was evaluated in vivo, showing excellent hemostatic, antibacterial, and healing-promoting effects in rat models of brain ventricle perforation and full-thickness skin defects infected with MRSA [70]. Two case reports on the use of antibiotic-loaded hydrogels for the treatment of PJI/MRSA-associated orthopedic device infections employed a local delivery approach (targeting the infection site to minimize systemic side effects and enhance therapeutic efficacy). The results demonstrated that hydrogels significantly increased local antibiotic concentrations and achieved sustained-release effects [71,72]. Hydrogels not only facilitate sustained drug release to prolong therapeutic half-lives but also allow for targeted delivery, rendering them highly suitable for the management of MRSA-PJI.

In the future, through continuous optimization of material design, exploration of underlying mechanisms, and collaborative progress in clinical translation, nanoparticle and hydrogel-based therapies will realize their full clinical potential in the fight against MRSA-PJI.

## 4. Discussion

PJI is a highly destructive complication following total joint replacement, with a high incidence and disability rate that not only severely impairs patients’ quality of life but also poses a significant challenge to clinical diagnosis and treatment [71]. Clinical practice has shown that the core issue in PJI treatment failure lies in the formation of biofilms by bacteria colonizing the surface of the joint prosthesis [72]. It is noteworthy that MRSA, one of the primary pathogens responsible for PJI, poses an even greater therapeutic challenge due to its intrinsic multi-drug resistance. This characteristic has made MRSA a focal point of concern in both orthopedic and microbiological research [47]. Currently, the traditional treatment approach for MRSA-PJI has clear limitations. Although surgical intervention remains essential to remove infectious foci, it is often associated with substantial tissue trauma and prolonged postoperative recovery. Moreover, the prevailing antibiotic strategies face multiple challenges: existing anti-MRSA agents are generally costly and exhibit notable side effects, which not only elevate the economic burden on patients but may also compromise clinical outcomes due to adverse drug reactions. The development process and marketing approval by regulatory agencies involve long timelines, making it difficult to rapidly meet clinical demands. Given these treatment limitations, this review explores new therapeutic strategies for MRSA-PJI, aiming to provide insights and references for overcoming current clinical bottlenecks and optimizing treatment regimens.

Monoclonal antibody therapy has already seen widespread application in the medical field. According to Umabs Antibody Therapy Database, China has 653 institutions engaged in monoclonal antibody drug development, ranking first in Asia and second globally, following the United States. To date, at least 212 monoclonal antibody therapies have been approved worldwide, benefiting millions of patients and demonstrating the transformative impact of monoclonal antibodies [73]. Moreover, the discovery of phage display technology has greatly accelerated the process of discovering therapeutic monoclonal antibodies. Phage display enables the identification of antibodies targeting nearly any target or epitope, including those that are toxic or non-immunogenic in animal immunity [74]. Amid the ongoing global COVID-19 pandemic, Ji Woong Kim used phage display to develop four specific human monoclonal antibodies targeting the receptor-binding domain of the SARS-CoV-2 spike protein [75]. Mary R. Ferguson utilized phage display to develop a monoclonal antibody targeting *Treponema pallidum* BamA protein, demonstrating superior activity [76]. Monoclonal antibodies, as specific agents against resistant bacteria, offer strong targeting capabilities with minimal side effects. As clinical research progresses, they are expected to play an increasingly important role in the treatment of various infections and diseases.

However, compared to small molecule drugs, monoclonal antibodies generally have low to moderate bioavailability, which may be due to their degradation by proteases in tissue fluids or the lymphatic system [77] (Table 1). Researchers could focus more on utilizing biomaterials such as nanoparticles and hydrogels for drug delivery, which would improve bioavailability and enhance drug stability. In such delivery systems, synergistic use of novel drugs and antibiotics could be achieved to improve therapeutic outcomes and reduce toxicity. Additionally, the high cost of monoclonal antibodies remains a challenge (Table 1). Future research may focus on phage expression systems, which could lower the cost of monoclonal antibodies through in vitro production, thus increasing the potential for clinical translation. This would help reduce the economic burden on patients and their overall quality of life.

Currently, research on phage therapy is progressing rapidly, with applications expanding into fields such as medical diagnostics, biological control, agriculture, nanotechnology, and drug discovery [78]. Phage therapy’s high specificity for target bacteria provides a promising new approach for treating MRSA-PJI. Claudia Ramirez-Sanchez et al. reported a successful case in which a two-stage exchange surgery for persistent methicillin-sensitive *Staphylococcus aureus* prosthetic joint infection was successfully treated following a second round of phage therapy [79]. The therapeutic application of phage therapy in MRSA-PJI is highly promising.

However, incorporating phage therapy into standard MRSA-PJI treatment protocols faces significant challenges (Table 1). The integration of genomics with machine learning-driven bioinformatics has already seen widespread use in cancer and other biomedical fields, and the latest advancements in machine learning-driven synthetic biology may further promote phage therapy as a viable clinical treatment option. Additionally, developing broad-spectrum phages is a potential solution. CRISPR technology could be used to engineer phages to expand their host range (e.g., knocking out receptor-binding protein genes). Immunogenicity also remains a concern, as the immune system could neutralize phages over time. Some researchers suggest engineering receptor-binding proteins and related domains through genomic techniques to redirect phage specificity and avoid resistance [80].

Although AMP therapy has shown several advantages for treating MRSA-PJI, it still faces limitations (Table 1). While AMPs have unique mechanisms of action, prolonged use may induce bacterial resistance (e.g., MRSA may resist peptides by upregulating efflux pumps or altering membrane phospholipid composition), necessitating monitoring of resistant strains and the development of new mechanisms targeting specific pathogens to reduce resistance risk [81,82]. AMPs also encounter challenges such as limited bioavailability and short half-lives. Researchers need to explore material science to address these issues, potentially by delivering AMPs through biomaterial carriers to improve bioavailability and extend half-lives [83,84]. For instance, designing nanoparticle carriers (e.g., liposomes or polymer micelles) for MRSA infections could enable targeted delivery of AMPs to the infection site, extending their half-life and improving bioavailability. High clinical translation barriers remain for AMP therapy. Researchers need to conduct more clinical trials to evaluate the safety and efficacy of AMPs for MRSA-PJI treatment. Overall, as emerging antimicrobial agents, AMPs offer distinct advantages and open new avenues for the management of infections.

Nanoparticles and hydrogels, as novel enhancement therapies, have already been widely applied across multiple therapeutic areas and have shown great potential in treating MRSA-PJI [85]. However, several challenges remain for these emerging therapeutic approaches, particularly the “delivery efficiency—safety—efficacy persistence” bottleneck. Liang et al. reported the development of VZZ-8 NPs, which in vitro studies demonstrated to effectively eradicate 93.84 ± 7.38% of MRSA and inhibit biofilm formation by 95.36 ± 0.13%. In a murine model of MRSA-induced PJI, VZZ-8 NPs exhibited robust antibacterial efficacy, concurrently suppressing local TNF-α and IL-6 expression, and preventing infection-induced osteolysis, highlighting their comprehensive therapeutic potential for PJI treatment [16]. Additionally, locally administered daptomycin-loaded nanoparticles were shown to sterilize infection sites in MRSA-infected rabbit osteomyelitis models after a single dose, with effective outcomes at both 4 and 14 days post-treatment [86]. Similarly, Boot et al. reported that a hyaluronic acid hydrogel loaded with gentamicin and vancomycin successfully eradicated chronic methicillin-resistant *Staphylococcus aureus* orthopedic infections in a sheep model through local administration [87]. Future research should focus on clinically relevant studies, including the development of MRSA-PJI animal models that closely mimic clinical conditions, and systematically evaluating the safety and antimicrobial efficacy of these therapies. This will pave the way for their clinical application.

The field of prosthetic joint infection management is at a pivotal juncture, with an unprecedented wave of clinical research challenging fundamental therapeutic assumptions and introducing novel treatment paradigms [88]. Currently, emerging technologies such as artificial intelligence (AI) are being widely applied across various fields. The development, optimization, and application of AI-enabled novel therapies may herald a promising direction for future therapeutic innovation. Researchers have already proposed machine learning-based methods that successfully predict AMPs within the global microbiome, identifying nearly a million prokaryotic AMP sequences and providing a rich resource for antimicrobial peptide synthesis [89]. With the integration of artificial intelligence, the design of hydrogels is undergoing significant transformation, driven by advances in human–machine interaction, machine learning, neural networks, and 3D/4D printing technologies [90]. Additionally, various biomaterials, including platelet-derived materials, plant fibers, and synthetic analogs of natural antimicrobial peptides, have demonstrated remarkable antimicrobial properties [91,92,93]. It is foreseeable that combining novel drugs with these biomaterials, together with the integration of advanced tools like AI, will provide more diverse and effective treatment options for MRSA-PJI. In addition, the novel therapies discussed in this article represent potential treatment strategies for a variety of diseases, including COVID-19 [94], cancer [95], and refractory bacterial infections [96], offering broad prospects for clinical application.

## Figures and Tables

**Table 1:** Mechanisms and Advantages/Disadvantages of Three Novel Drugs.

Treatment Strategy	Mechanism	Advantages	Disadvantages
Monoclonal Antibodies	Specific antibodies targeting resistant bacteria	Strong specificity, low side effects	High production cost, low bioavailability
Phage Therapy	Utilizing viruses to directly kill resistant bacteria	Strong targeting, effective against resistant bacteria	Narrow host range, potential for immune rejection
Antimicrobial Peptides	Disrupting cell membranes or cell walls leading to cell lysis	Low toxicity, strong thermal stability	Long-term use may induce bacterial resistance, Poor bioavailability, short half-life

## Data Availability

No new data were created or analyzed in this study.

## Abbreviations

The following abbreviations are used in this manuscript:

TJR	Total joint replacement
PJI	Periprosthetic Joint Infection
MRSA	Methicillin-Resistant *Staphylococcus Aureus*
PBP2a	The mec gene on the Staphylococcus chromosome
MRSA-PJI	Periprosthetic Joint Infection caused by Methicillin-Resistant *Staphylococcus Aureus*
mAbs	Monoclonal Antibodies
IgG	Gamma Immunoglobulin
Fab	Antigen-Binding Fragment
Fc	Crystallizable Fragment
AMP	Antimicrobial Peptides
MPS	mononuclear phagocyte system
PSA	Poly-sialic acid
AI	artificial intelligence
